# Supplementary material for: Validation of a rapid, saliva-based, and ultra-sensitive SARS-CoV-2 screening system for pandemic-scale infection surveillance
Source: Sci Rep. 2022 Apr 8;12:5936. doi: 10.1038/s41598-022-08263-4 (PMC8990279; doi:10.1038/s41598-022-08263-4)
Supplement: Supplementary file 1 — Supplementary Information. [file 41598_2022_8263_MOESM1_ESM.docx]

Supplementary Materials for

Validation of a rapid, saliva-based, and ultra-sensitive SARS-CoV-2 screening system for a pandemic-scale infection surveillance

**Authors:** Robert E Dewhurst^1,2^, Tatjana Heinrich^1,2^, Paul Watt^2,3^, Paul Ostergaard^2^, Jose Maria Marimon^4^, Mariana Moreira^5^, Philip E Houldsworth^5^, Jack D Rudrum^1,2^, David Wood^6^ and Sulev Kõks^1,7,8*^

**Affiliations:**

^1^Perron Institute for Neurological and Translational Science; Perth, 6009, Western Australia, Australia.

^2^Avicena Systems Ltd, West Perth, 6005, Western Australia.

^3^Telethon Kids Institute, University of Western Australia, Perth, 6009, Western Australia.

^4^ Biodonostia Health Research Institute, Infectious Diseases Area, Osakidetza Basque Health Service, Donostialdea Integrated Health Organization, San Sebastián, Spain.

^5^Lancs Lamp Laboratory, Heatley House, Bowran Street, Preston, PR1 2UX, United Kingdom.

^6^University of Western Australia; Perth, 6009, Western Australia.

^7^Centre for Molecular Medicine and Innovative Therapeutics, Murdoch University, Perth, 6150, Western Australia.

^8^Prion Ltd, Tartu, 50410, Estonia.

*Corresponding author. Email: [sulev.koks@perron.uwa.edu.au](mailto:sulev.koks@perron.uwa.edu.au)

# This PDF file includes:

Suppl. Figures. S1 to S3 and Suppl table S1


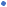

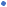

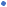

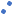

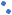

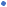

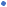

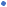

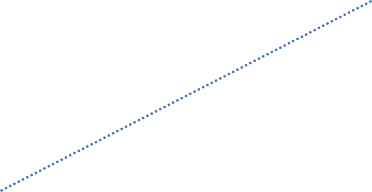


25

RT-LAMP versus RT-qPCR on extracted RNA from clinical saliva samples: Zhang E1 primers

20

R² = 0.5067

15

10

20

25

30

35

40

45

Ct


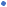

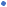

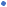

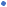

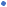

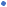

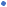


RT-LAMP versus RT-qPCR on extracted RNA from clinical

saliva samples: Novacyt-S primers

25

R² = 0.6111

20

15

10

20

25

30

Ct

35

40


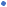

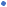

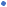

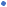

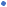

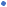

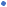

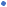


RT-LAMP versus RT-qPCR on extracted RNA from clinical saliva samples: Huang O117 primers

15

R² = 0.7361

10

5

20

25

30

Ct

35

40


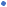

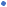

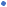

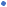

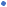

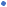

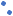

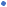


RT-LAMP versus RT-qPCR on extracted RNA from clinical saliva samples: Zhang E1/N2 primers

25

R² = 0.9064

20

15

10

20

25

30

Ct

35

40

TTT (min)

TTT (min)

TTT (min)

TTT (min)

**Suppl. Figure S1: Concordance between RT-LAMP and RT-qPCR**. There was a proportional relationship between Ct values (RT-qPCR) and TTT values for five published RT-LAMP primer sets, demonstrating that RT-LAMP provides a quantitative readout of viral load.


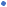

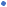

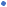

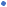

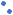

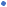

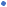


20

RT-LAMP versus RT-qPCR on extracted RNA from clinical saliva samples: Huang S17 primers

R² = 0.7821

15

10

5

20

25

30

Ct

35

40

TTT (min)

**A**

**B**

S**uppl. Figure S2. A. Detection of Twist SARS-CoV-2 control RNA 23 (“Delta variant”), and B. Detection of Twist SARS-CoV-2 control RNA 48 (“Omicron variant”).** RT- LAMP (Hayat Genetics/Avicena) was performed in a fluorescence mode on a dilution series of Twist Bioscience Control 23 and 48 synthetic RNA. Each datapoint is the mean of four technical replicates. Error bars represent the standard deviation.

**Suppl. Figure S3. Comparative analysis of NIBSC standardised viral load panel between Hayat and Optigene Direct RT-LAMP assays**

**Suppl. Table S1. Detection of the other pathogens by the RT-LAMP test.**

| Sample | Respiratory Pathogen | Hayat LAMP (n=4) |
| --- | --- | --- |
| MRES01 | *Human Metapneumovirus* | *not detected* |
| MRES02 | *Legionella pneumophila* | *not detected* |
| MRES03 | *Adenovirus* | *not detected* |
| MRES04 | *Rhinovirus/ Enterovirus, Picornavirus, Enterovirus* | *not detected* |
| MRES05 | *Parainfluenza 2 & group* | *not detected* |
| MRES06 | *Coronavirus, Coronavirus OC43, RSV, RSV-B* | *not detected* |
| MRES07 | *Bordetella species, Bordetella pertussis, Picornavirus, Rhinovirus, Enterovirus* | *not detected* |
| MRES08 | *Parainfluenza 4 & group* | *not detected* |
| MRES09 | *Chlamydophila pneumoniae* | *not detected* |
| MRES10 | *Human Metapneumovirus* | *not detected* |
| MRES11 | *No Pathogen Detected* | *not detected* |
| MRES12 | *Parainfluenza 1 & group* | *not detected* |
| MRES13 | *Mycoplasma pneumoniae* | *not detected* |
| MRES14 | *No Pathogen Detected* | *not detected* |
| MRES15 | *Parainfluenza 3 & group* | *not detected* |
| MRES16 | *Coronavirus, Coronavirus NL63* | *not detected* |
| MRES17 | *Adenovirus, Influenza A, Influenza A H3* | *not detected* |
| MRES18 | *Picornavirus, Rhinovirus, Rhinovirus/ Enterovirus* | *not detected* |
